# Habitat Differences in Resource Density and Distribution Affect Ecology and Life History of a Landscape‐Modifying Fish

**DOI:** 10.1111/mec.70145

**Published:** 2025-10-13

**Authors:** Aneesh P. H. Bose, Boyd Dunster, Jonathan Henshaw, Lukas Koch, Jacqueline Grimm, Kristina M. Sefc, Alex Jordan

**Affiliations:** ^1^ Department of Wildlife, Fish and Environmental Studies Swedish University of Agricultural Sciences (SLU) Umeå Sweden; ^2^ Behavioural Evolution Research Group Max Planck Institute of Animal Behavior Konstanz Germany; ^3^ Centre for the Advanced Study of Collective Behaviour University of Konstanz Konstanz Germany; ^4^ Institute of Biology University of Freiburg Freiburg Germany; ^5^ Institute of Vertebrate Biology Czech Academy of Sciences Brno Czechia; ^6^ Institute of Biology University of Graz Graz Austria

**Keywords:** ecological niche, environmental heterogeneity, evolutionary niche, genetic parentage, reproductive success, sexual selection

## Abstract

Resource heterogeneity is a widespread phenomenon, as resources are rarely spaced evenly across a landscape. Variation in resource density and distribution can have a myriad of behavioural, ecological, and evolutionary consequences for populations, yet clarifying these effects is still challenging. We combine both novel and previously published data on genetic parentage, relatedness, life history, and predation to present a comprehensive field study of a shell bed in Lake Tanganyika. Here, a wild population of the cichlid fish *Neolamprologus multifasciatus* is naturally subdivided into habitat regions that differ immensely in shelter density and distribution, as well as in the capacity for the fish to physically rearrange their shelters into clusters (i.e., engage in niche construction). Shelters were evenly, densely, and continuously spaced in one habitat, while they were highly clustered in the other habitat. We expected the environmental potential for polygyny to be greater in the clustered habitat relative to the continuous habitat. Predation regimes and life history traits differed, with *N. multifasciatus* in the evenly distributed habitat experiencing higher predation threats, earlier maturation, and slower growth than those in the clustered habitat. Metrics of selection, however, were surprisingly consistent between the two habitats, as were patterns of dispersal. Overall, our research leverages the natural subdivision of a wild population into distinct habitats to investigate the ecological and evolutionary implications of resource heterogeneity and habitat modification.

## 1 Introduction

Heterogeneous environments are ubiquitous in nature and characterised by uneven distributions of resources and biotic or abiotic conditions. Spatial heterogeneity of resources, such as food, shelter, or mates, can affect many aspects of animal behaviour and life history. For example, compared to environments with even food distributions, heterogeneity in food resources can attract more foragers to higher‐quality patches, but also incite competition among them with downstream costs for their reproductive success (Trevail et al. 2019). Dispersal strategies may also differ between heterogeneous and homogeneous environments, if these environments offer different motivations to depart a natal territory or affect the ease of navigating throughout the landscape (Clobert et al. 2009). Mating systems too can be affected by the degree of resource heterogeneity, particularly if dominant individuals are able to competitively exclude rivals from accessing resource patches (Emlen and Oring 1977). It is often assumed that the arrangement of resources in an environment is determined by processes beyond the control of the organisms that use those resources.

However, organisms that niche construct can exert their own influence on their environment by altering, for example, the physical or chemical structure of their surroundings (Laland et al. 2016). Making changes to their ecological surroundings and to the environmental factors that affect them, niche constructors alter their so‐called “evolutionary niche”, i.e., the sum of the selection pressures their population faces (Odling‐Smee et al. 2013; Trappes 2021). By making habitat modifications, organisms induce changes that can have numerous consequences, both ecological (by altering the interactions that occur within and between species or populations) and evolutionary (by altering the selection pressures that lead to fitness differences within a population). Through their actions, niche‐constructing organisms can also increase or decrease resource heterogeneity in the environment, and thereby influence processes that respond to resource distribution. Matthews et al. (2014) lay out three criteria for niche construction to shape evolution. First, an organism must significantly modify its environmental conditions. Second, the organism‐mediated environmental modifications must influence selection pressures (either on the niche constructor itself or on other organisms). Third, there must be an evolutionary response in at least one recipient population caused by the environmental modification. The broad definition of niche construction necessarily means that all organisms could conceivably be considered niche constructors, because it simply requires that organisms alter selection pressures acting on themselves or others through environmental modification no matter how small (Aaby and Ramsey 2022; Laland et al. 2016). This ubiquity makes it crucial to highlight cases of niche construction that have biologically meaningful effects (Matthews et al. 2014), whether these effects are on patterns of selection (for niche construction to count as a mediator of evolutionary change) or on trait expression more generally.

**FIGURE 1:**
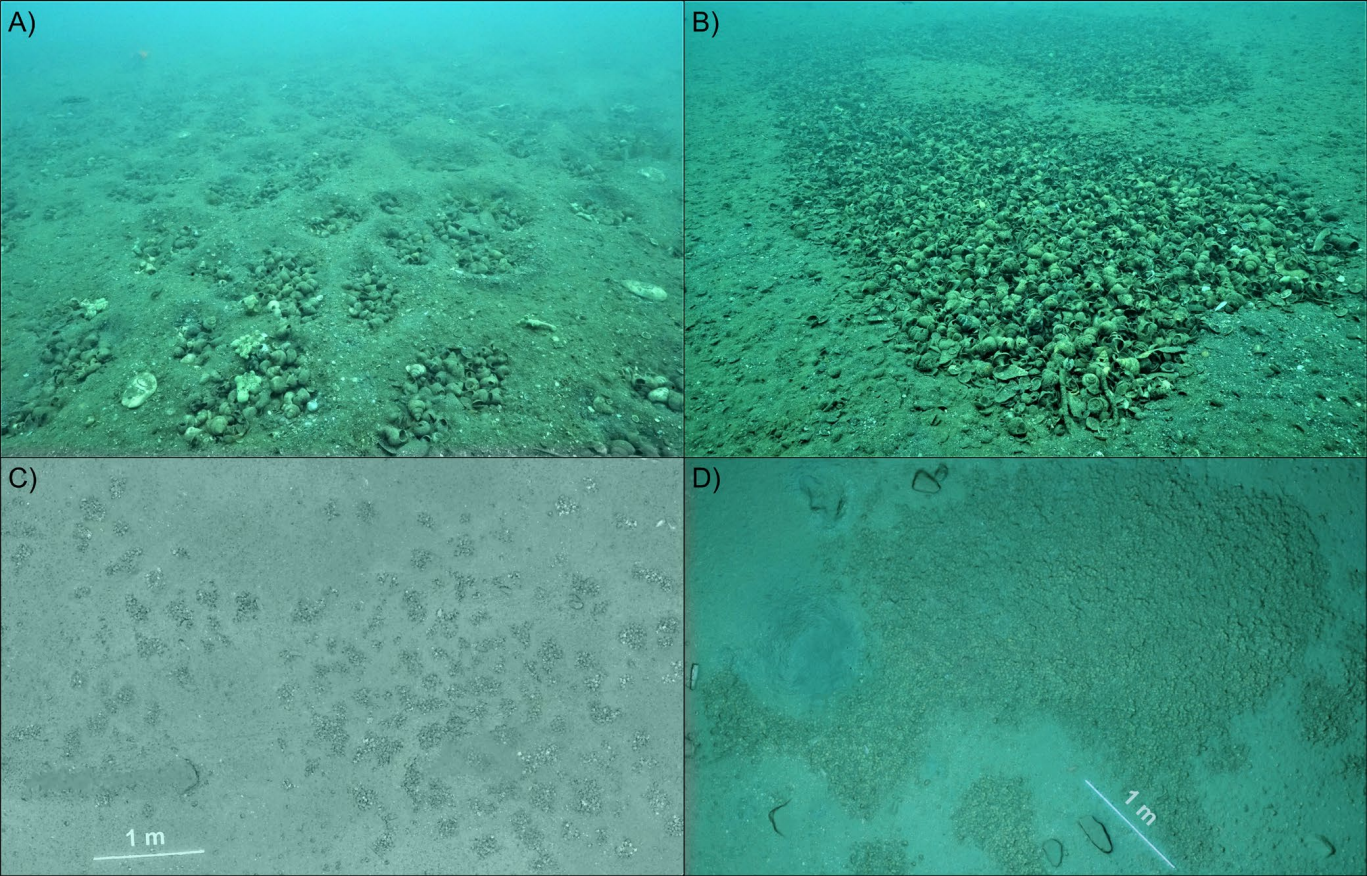
Examples of a ‘clustered’ (A) and a ‘continuous’ (B) region of the shell bed at the Mutondwe Island shell bed in Lake Tanganyika. Maps, created with Structure‐from‐Motion photogrammetry, of the clustered (C) and continuous (D) study areas sampled for population genetics in 2019 and 2023 respectively. Each cluster of shells in (C) is the territory of a separate *N. multifasciatus* social group. The continuous region in (D) is composed of a bare layer of shells with little sand. Note the differences in shell density and distribution between the two regions. Photos and maps are by Aneesh Bose.

In this field study, we conducted comprehensive population surveys and microsatellite genotyping for genetic parentage and relatedness analysis to explore the relationships between resource heterogeneity and metrics of selection, dispersal, predation pressure, and life history traits in a cichlid fish that lives in two habitats in Lake Tanganyika, one of the Rift Valley lakes in East Africa. Certain regions of the lake floor, termed ‘shell beds’, are covered by massive accumulations of empty snail shells which are home to a unique assemblage of fishes and invertebrates (Salzburger et al. 2014). Shell beds can be divided into two habitat types that differ principally in resource density and distribution—and while one type of shell bed is modifiable by the fish that live there, the other is not. Both types of shell bed are home to *Neolamprologus multifasciatus* , which lives in long‐term social groups on fixed territories where each fish digs in the sand to excavate snail shells that they use as shelters and breeding chambers. Each fish resides within one ‘home’ shell (Bose et al. 2024). The scooped‐up sand is then deposited along the borders of their group's territory, effectively building walls between themselves and neighbouring groups. Such actions produce a visually striking pattern on the sandy lake floor, restructuring it from a homogenous distribution of shells to a cratered landscape, with each crater containing a territory and a discrete cluster of shells. We refer to this habitat as the ‘clustered shell bed’ (Figure 1A). The other type of shell bed where *N. multifasciatus* lives is a continuous layer of shells and shell fragments with little sand. *Neolamprologus multifasciatus* are too small to move shells or to dig in these locations, and so the homogenous distribution of shells in these areas cannot be altered—no sand walls can be constructed between neighbouring territories. We refer to this habitat as the ‘continuous shell bed’ (Figure 1B).

These two types of shell beds can be situated next to, or even interspersed with, each other such that the lake floor is a patchwork of continuous and clustered beds, with each patch spanning several to tens of meters across. Given their proximity, the fish living in each of these bed types experience the same overarching lake conditions and differ only in the abundance of sand and therefore in their ability to dig and erect sand walls. Furthermore, dispersal distances are extremely limited (typically less than two meters, Bose, Koch, et al. 2022), far smaller than the size of individual regions of continuous or clustered shell beds; with such highly constrained dispersal, most fish will likely spend their entire lives within one type of shell bed. Thus, wild populations of *N. multifasciatus* are subdivided into natural replicates that provide an excellent test bed to study how differences in resource heterogeneity affect patterns of selection, dispersal, and trait expression.

By clumping shells together and surrounding them with sand walls, *N. multifasciatus* in the clustered shell bed may be using niche construction to influence their own ‘environmental potential for polygyny’ (*sensu* Emlen and Oring 1977). This foundational concept in mating systems evolution posits that when critical resources are spatially clumped and more easily defendable, there is greater opportunity for a small, competitive subset of the population to monopolise a large proportion of the available resources. In so doing, the scope for multiple mate monopolisation also increases, thereby increasing the overall strength of sexual selection. In Lake Tanganyika, niche construction through sand digging is the process by which shell‐dwelling cichlid fishes like *N. multifasciatus* transform the sandy lake floor into clustered shell beds—areas with a high environmental potential for polygyny. In parallel, on continuous shell beds where it is not possible to construct sand barriers between territories, the environmental potential for polygyny is expected to remain low. Aggression between *N. multifasciatus* declines sharply as the distance separating neighbours and their respective shells increases, likely because *N. multifasciatus* are highly susceptible to predation if they venture too far from their home shells (e.g., within‐group aggression drops markedly as fish's home shells become separated by more than 10–15 cm, Bose et al. 2023). Therefore, by helping to maintain physical separation between neighbours we expected sand walls to make shell clusters more defendable and monopolizable by dominant individuals. However, for a high environmental potential for polygyny to affect mating systems and sexual selection, individuals must be able to capitalise on the clumped nature of their resources (Emlen and Oring 1977). Based on our previous work in the clustered shell bed, we have shown that large dominant males aggressively control territories around each cluster of shells; they use their shell clusters to attract females from neighbouring territories (Jordan et al. 2016) and then monopolise reproduction with female group members (Bose, Dabernig‐Heinz, et al. 2022). Females also defend multiple shells within their group's territory from rival females (Bose et al. 2021). We hypothesized that the inability to construct sand walls on the continuous shell bed would lead to a breakdown of the ability of dominant individuals to control access to mates and resources. To test this, we combined previously published data on reproductive skew among *N. multifasciatus* in a clustered shell bed with new complementary data from a continuous shell bed. We predicted that the strength of (sexual) selection in the clustered shell bed would be higher relative to the continuous shell bed.

The distribution of resources (shells) in each shell bed represents the backdrop against which the fish interact with each other and respond to threats in their environment. We therefore additionally investigated whether *N. multifasciatus* living in either type of shell bed differed in patterns of dispersal, predation risk, and life history. Vacant shells across the landscape represent locations to disperse into or to take shelter from predators when *en route* while dispersing; when there are few viable dispersal destinations within the typical dispersal range, i.e., two meters, then the fish's already limited dispersal becomes even more constrained (Bose, Koch, et al. 2022). We investigated dispersal patterns in a continuous shell bed relative to a clustered bed; while greater densities of shells in continuous beds might facilitate dispersal by offering more shelters as ‘stepping stone’ refuges, any residents in those shells could also hinder dispersal. We also predicted that predators would spend more time hunting on continuous beds than clustered beds because the greater density of shells provides more living space for numerous shell bed residents, including fishes, shrimp, and crabs (each individual of which typically occupies a single shell, Bose et al. 2024). In relation to the density of *N. multifasciatus* , we expected predation pressure to be higher on continuous shell beds relative to clustered shell beds. Finally, we also predicted that without sand walls to buffer against neighboring competitors, and potentially also having to face higher predation pressures, *N. multifasciatus* living on continuous shell beds would display ‘faster’ life histories. In particular, we expected the fish on continuous beds to mature earlier and invest less in somatic growth than those on clustered beds (Bonduriansky et al. 2008; Magnhagen 1991).

Here, we leverage a rare opportunity where a wild population of fish is naturally subdivided into habitats that differ in resource density and distribution and are either modifiable or not modifiable. We first tested whether *N. multifasciatus* influences their own ‘environmental potential for polygyny’ (*sensu* Emlen and Oring 1977) through niche construction via comprehensive genotyping and parentage analyses. We compared metrics of selection between a subpopulation living on a clustered patch where the fish form discrete shell clusters and another subpopulation living on a continuous patch where they live on a dense, homogeneous distribution of shells. Furthermore, given the far‐reaching effects of resource heterogeneity on many ecological and evolutionary processes, we also examined whether fish living in the two habitat types differed in dispersal, predation pressure, and life history trait expression.

## 2 Methods

During field trips to southern Lake Tanganyika from August to October of 2019 and 2021, and from April to May of 2023 and 2024, we studied a shell bed (near Mutondwe Island, Zambia, 8°42′48.8″S, 31°07′23.3″E), which contains both clustered and continuous patches. Over the course of these field seasons, we sampled *N. multifasciatus* individuals living in either shell bed type for population genotyping, parentage and relatedness analyses, life history measurements, and predator surveys.

### 2.1 Description of Shell Bed Field Sites and General Sampling Methodology

In 2019, we conducted intensive population genetics sampling of all *N. multifasciatus* living on a patch of clustered shell bed 10–11 m deep that was approximately 15.1 m^2^ in area (Figure 1C). All field sampling was performed while on SCUBA. In 2023, we followed an identical procedure to sample all *N. multifasciatus* living on a patch of continuous shell bed 16 m deep that was approximately 7.4 m^2^ in area (Figure 1D). Because shell‐dwelling fish hide within their shells when approached by a diver, each fish could be collected by hand by picking up their shells. The fish were extracted from their shells (following methods in Bose et al. 2023) and sedated with clove oil. The fish were then sexed by inspection of their urogenital papilla, measured for standard length (mm, SL), and recorded as either adult or juvenile based on the presence of distinct banding along the sides of their body denoting sexual maturity (Kohler 1998). When juvenile fish are in the process of transitioning to maturity, faint bands can already be noticed on their sides before the pattern develops fully, and so we used the presence of faint banding to record ‘size‐at‐maturity’ for fish in this transitionary phase. Fish that were larger than 1.6 cm were fin‐clipped on their anal fins (taking at most 2 × 2 mm of tissue) and then returned with their shells to where they were captured, when fully recovered from sedation. Fish smaller than or equal to 1.6 cm were euthanized with an overdose of clove oil (Neiffer and Stamper 2009) and sampled whole due to the relatively large amount of tissue that clipping would have removed. All fin clips or whole fish were stored in 99% ethanol for later genetic analysis. As our sampling progressed in each shell bed patch, we would periodically return to already‐sampled areas to check for missed, unclipped fish, which became exceedingly rare, lending high confidence that we had sampled (nearly) all individuals living in each patch. Data from the 2019 clustered shell bed patch have since been published in (Bose et al. 2023; Bose, Dabernig‐Heinz, et al. 2022; Bose, Koch, et al. 2022), but are re‐analysed here to answer new research questions.

Across the various field seasons, we also visited other clustered and continuous patches on the shell bed to (i) take measures of adult body size and size‐at‐maturity, (ii) survey the community of heterospecifics active at each patch, and (iii) capture a set of adult males from both shell bed types for age analysis using their saccular otoliths. Note that the physical structure of the shell bed is rather invariant over time (AB, BD, LK, AJ personal observations that areas of the shell bed that are either clustered or continuous have consistently remained so for over 10 years of routine field work at this lake site). In fact, maps of two continuous shell beds at our field site reveal a highly consistent spatial layout across a one‐year timespan (Figure S1).

### 2.2 Genetic Parentage and Sexual Selection Differences Between the Clustered and Continuous Shell Beds

We collected fin clips from 835 fish (239 adult females, 191 adult males, 405 juveniles/offspring) from the clustered bed in 2019 (Figure 1C), and from 466 fish (123 adult females, 90 adult males, 253 juveniles/offspring) from the continuous bed in 2023 (Figure 1D). Both sampled regions of the shell bed were encircled by a stretch of open sand (estimated as > 1 m in width for the clustered region and > 0.5 m for the continuous region), representing a sizeable dispersal barrier for *N. multifasciatus* (see Bose, Koch, et al. 2022). These regions were chosen specifically because these stretches of open sand would help ensure that the mating patterns we uncovered in each region were generated only from individuals living there.

DNA was extracted from each tissue sample using a standard Chelex protocol (Walsh et al. 1991), and all individuals were genotyped at 20 microsatellite loci divided into three multiplexes following the methods outlined in Bose, Dabernig‐Heinz, et al. (2022). Marker polymorphism was estimated in population samples consisting of 238 fish from the clustered shell bed and 90 fish from the continuous shell bed (including both males and females captured from across each shell bed to reduce the influence of population kinship structure on population allele frequency estimations (Bose, Dabernig‐Heinz, et al. 2022)). Average expected heterozygosity across loci was 0.75 for the clustered shell bed and 0.74 for the continuous shell bed. All loci complied with Hardy–Weinberg equilibrium expectations after correction for multiple testing (see also Tables S1 and S2 for marker polymorphism indices). Using the multilocus genotypes from each fish and the software *Cervus* (version 3.0.7; Kalinowski et al. 2007), we identified candidate parents of juveniles collected within each shell bed type. We only considered individuals that were successfully genotyped at ≥ 15 loci, which excluded 65 fish from the clustered shell bed (52 juveniles, 11 females, and 2 males). We detected at least one parent for 92% of the offspring analysed in the clustered shell bed and for 73% of the offspring analysed in the continuous shell bed.

All analyses were conducted in R (version 4.4.3, R Core Team 2025). Using the genetic parentage data from these two shell beds, we calculated the number of genetic mates for each adult as a measure of their mating success (MS) and the number of offspring assigned to them by genetic parentage analysis as a measure of their reproductive success (RS). Note that because we analyze the parentage of young fish that comprise a variety of ages before they reach sexual maturity, our scores of reproductive success will likely include a combination of sexual selection acting on the parents themselves and natural selection acting on the offspring's phenotypes (Anthes et al. 2017). We then calculated relative MS and RS by mean‐scaling MS and RS for each sex and shell bed separately. This was done because Anthes et al. (2017) emphasizes the use of relative, rather than absolute, measures of reproductive success and mating success when comparing metrics of sexual selection across populations that differ in, for example, mean sex ratios, mating success, or offspring production. To test whether fish living on the clustered shell bed experience more intense sexual selection relative to the fish living on the continuous shell bed, we calculated several metrics of sexual selection (as well as selection more generally) and compared them between shell beds for each sex. We calculated the opportunity for selection (*I*) as the variance in relative RS, the opportunity for sexual selection (*I*s) as the variance in relative MS, and the Bateman gradient (*β*ss) as the regression coefficient of relative RS on relative MS from an ordinary least squares regression. We also calculated the selection gradient for body size (*β′*_size_) as the regression coefficient of raw RS on adult standard length (*z*‐scored) from a negative binomial generalized linear model (GLM). We ran one GLM per sex, in which body size, shell bed type, and their interaction were included as predictors (using the ‘glmmTMB’ R package, Brooks et al. 2017, assuming a ‘nbinom2’ family). The model coefficients for the effects of standard length on RS for each shell bed type were extracted and then mean‐scaled by their respective shell bed population's average RS. Note that we used raw, not relative, RS in these GLMs to preserve the integer responses required by count models; however, we still applied a mean‐scaling to be able to compare *β′*_size_ between shell bed types.

To compare the metrics of selection between the shell bed types, we used a series of permutation tests. Specifically, we took the observed difference in *I*, *I*s, *β*ss, and *β*′_size_ between the two shell bed types (for males and females separately) and compared it to a null distribution built by permuting the shell bed labels 10,000 times.

### 2.3 Dispersal Patterns in the Clustered and Continuous Shell Beds

We recorded the precise locations of where fish were captured in both the clustered and continuous shell beds (this was done for 90.0% of the males, 90.2% of the females, and 77.5% of the juveniles in the continuous shell bed, and 100% of all adults and juveniles in the clustered shell bed). We took downward‐facing video footage of each study area (with GoPro cameras set to ‘linear’ field of view) and used Structure‐from‐Motion photogrammetry (Westoby et al. 2012) to recreate the spatial layout of each site (Figure 1C,D). From these maps, we calculated the pairwise distances between all *N. multifasciatus* individuals for which we had spatial information based on their Cartesian coordinates as placed in ImageJ (v 1.53e).

We then correlated spatial separation between individuals to their genetic relatedness. We used the R package “Demerelate” (Kraemer and Gerlach 2017) to calculate symmetric Lynch–Ritland pairwise relatedness estimators (rLR, Lynch and Ritland 1999) for each pair of fish in our dataset that had at least 10 microsatellite loci successfully genotyped (which was 97.9% of all females and 99.0% of all males in the clustered bed, and 100% of all males and females in the continuous bed). We fit two generalised additive mixed models (GAMM) assuming a Gaussian error distribution, one to investigate how male–male relatedness varies with spatial separation and another for female–female relatedness. We first applied a Yeo–Johnson power transformation to the rLR values to improve the normality and symmetry of the model residuals (Yeo and Johnson 2000). We included shell bed type as a predictor variable as well as geographic distance between fish (in cm) as a cubic regression spline composed of five knots. As rLR estimates involve dyads of fish, and each fish contributes to multiple rLR estimates in our dataset, we also included random slopes for ‘fish1 ID’ and ‘fish2 ID’ over geographic distance in the models.

### 2.4 Predation Regime Differences Between the Clustered and Continuous Shell Beds

During the 2024 field season, we quantified the presence of heterospecific predators that were active at each of the two shell bed types. On five separate mornings, around 9:00 AM, we set up a pair of video cameras (GoPro Hero 10 Black, set to a ‘wide angle’ FOV) at different locations across the shell bed, facing horizontally out across the shells. This camera orientation allowed us to record the various pelagic and demersal fish that spent time just above the lake floor. On each day, one camera recorded at a clustered shell bed region while the other recorded at a continuous shell bed region, with entirely new regions being chosen each separate day. On each day, the chosen clustered and continuous shell bed regions were always near one another (< 20 m apart) but not overlapping in the cameras' views. Pairing the cameras in this way ensured that we controlled for depth differences in predator community (cameras were matched for depth within pairs, and depths of the camera pairs ranged from ~10–18 m) and also controlled for daily differences in turbidity and visibility, such that each camera within a pair overlooked an equivalent patch area (visibility was estimated to be 8–10 m each day). Each camera recorded for ~1 h, and we omitted the first 5 min after camera setup to reduce any effects of diver presence on the community of fishes swimming above each shell bed region. The recordings were watched and scored for the presence of four common predatory species, *Lepidiolamprologus elongatus* , *L. attenuatus* , *L. cunningtoni* , and *N. tetracanthus* , as well as *Lobochilotes labiatus* . *L. labiatus* is a zoobenthos feeder with a specialised mouth morphology for sucking small invertebrates from crevices and shells (Takeyama 2021). The foraging activity of large *L. labiatus* is highly disruptive to the shell bed, as their suction feeding overturns and rearranges the shells, which can in turn attract attention from other hunting species nearby (AB, BD, LK, AJ personal observations). The time durations (in seconds) when at least one individual of each of these species was observed above the shell beds were scored.

We fit a general linear mixed effects model to these data to test whether the continuous and clustered shell beds differed with respect to the proportion of time that each heterospecific was present on the beds. The time duration (in seconds, after applying a log (x + 1) transformation) that at least one member of each species was observed on the bed was used as the response variable. We included type of shell bed (clustered vs. continuous), category of heterospecific (predatory species vs. *L. labiatus* ), and their interaction as predictor variables. The log of the time duration that each video was scored was included as a model offset. We also included species nested within day as a random intercept. Here, ‘day’ accounted for the fact that each pair of cameras was set at the same time and general place in the broader shell bed. Finally, we used ‘emmeans’ to make pairwise contrasts between the two shell bed types.

### 2.5 Life History Differences Between the Clustered and Continuous Shell Beds

As described above, we comprehensively sampled all *N. multifasciatus* living on one clustered and one continuous shell bed during the 2019 and 2023 field seasons respectively. To supplement these samples, we visited three additional clustered shell beds in the 2021 and 2023 field seasons (the data from which are included in Bose et al. 2024 but re‐analyzed here). In total, we sexed and measured the standard length of 772 adult *N. multifasciatus* from these sampling trips (431 females, 341 males). We also recorded size‐at‐maturity for 107 juveniles living in these regions, using the presence of faint banding patterns on the sides of their bodies to recognize maturing individuals (41 fish from the continuous shell bed and 66 fish across the clustered beds). Lastly, in 2023 we used an overdose of MS‐222 to euthanize nine adult males from the continuous shell bed and 10 adult males from different social groups in the three additional clustered shell beds. These males were measured for standard length (to the nearest mm), weighed (to the nearest 0.001 g), and had their saccular otoliths extracted, cleaned, and stored dry for later age analysis.

#### 2.5.1 Adult Body Size Differences Between the Clustered and Continuous Shell Beds

We first tested whether average adult body sizes differed between the shell beds using a linear mixed effects model (LMM). We included fish standard length as the response variable, and sex (categorical: male, female) and shell bed type (categorical: clustered, continuous) as predictor variables. We also included a random intercept capturing each combination of year and shell bed region that we sampled. We then used the ‘emmeans’ R package (Lenth 2023) to carry out *post hoc* comparisons. Next, we tested whether the *upper limits* of adult body sizes differed between shell bed types. To do this, we calculated the 90th percentile of standard length as an estimate of a ‘large’ individual for each sex in each shell bed type. We then bootstrapped 95% confidence intervals around these estimates and assessed whether they overlapped with one another.

#### 2.5.2 Size‐At‐Maturity Differences Between the Clustered and Continuous Shell Beds

*N. multifasciatus* that are sexually mature can be sexed by inspection of their urogenital papillae, but this approach is less reliable for immature fish. Thus, we refrained from directly assigning sex to the juveniles with faint banding that were transitioning to maturity. The body size distributions of these transitioning fish, however, showed clear bimodality consistent with females maturing at a smaller size than males (Kohler 1998). We therefore implemented gaussian mixture models (using the ‘flexmix’ R package, Gruen and Leisch 2023), assuming that the body size data from each shell bed type were comprised of two distinct Gaussian distributions, one for maturing males and another for maturing females. With these mixture models, we estimated the mean and variance parameters for male and female size‐at‐maturity for each shell bed.

#### 2.5.3 Size‐At‐Age Differences Between the Clustered and Continuous Shell Beds

Otolith aging was conducted at the Department of Aquatic Resources at the Swedish University of Agricultural Sciences on the 19 adult males collected from the clustered and continuous shell beds. Saccular otoliths were thin‐sectioned, and then growth rings were counted using light microscopy. Note that there have been no otolith aging validation studies for *N. multifasciatus* , but growth rings corresponding to annuli have been verified in another cichlid species from the same southern region of Lake Tanganyika (*Tropheus moori*, Egger et al. 2004). Aging was done blind to the origins of each fish. We then used linear regression models to test whether size‐at‐age differed between the two shell bed types.

### 2.6 Ethics Statement

All methods adhered to the ASAB/ABS Guidelines for the Use of Animals in Research. Fieldwork was carried out with approval from the Fisheries Department at the Ministry of Fisheries and Livestock Zambia, under study permits issued by the government of Zambia (No. G7067690 and C3195368, SP260718/7‐21, SP365210/6‐23, SP425444/4‐24) and in conjunction with a memorandum of understanding with the University of Zambia (MOU 101/14/11). The study species is listed as ‘Least Concern’ on the IUCN Red List of Threatened Species.

## 3 Results

### 3.1 No Clear Differences in Selection Metrics Between Shell Beds

**FIGURE 2:**
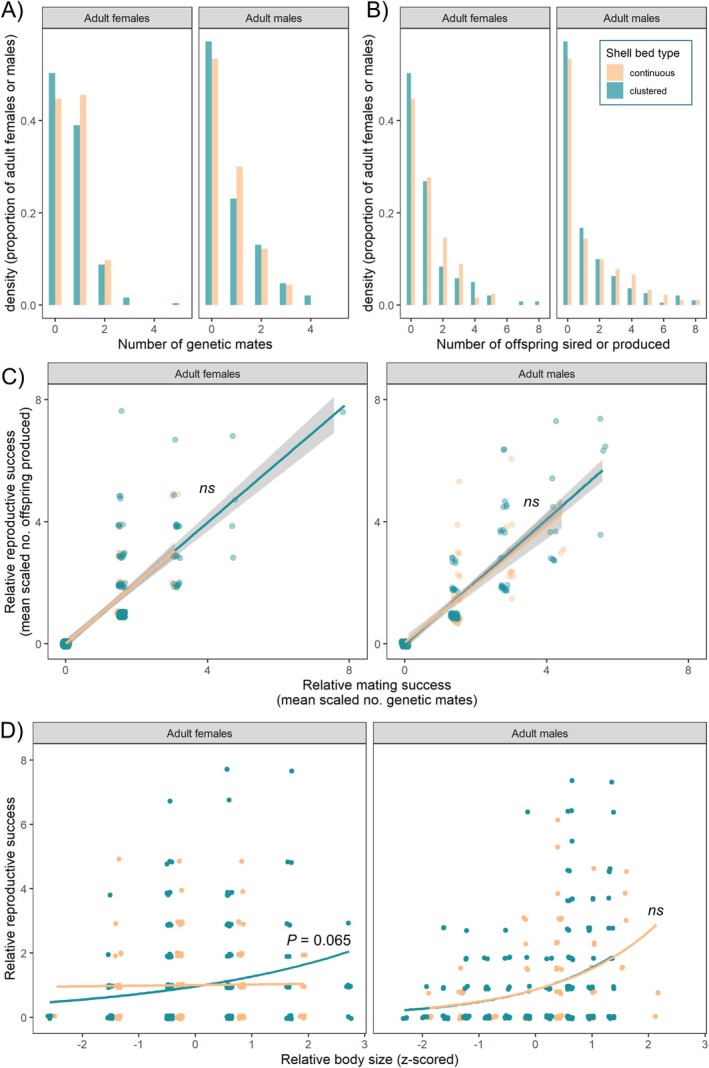
Density histograms for (A) mating success and for (B) reproductive success given for adult females and males in the clustered as well as the continuous shell bed (*N*_total_ = 643 adults). (C) Bateman gradients, *β*ss, visualised for each sex and shell bed type. (D) Selection gradients for body size, *β′*_size_, visualised for each sex and shell bed type.

Mating and reproductive success were reconstructed from genetic parentage data as the number of mates and the number of offspring, respectively. Male mating success ranged from 0 to 4 female mates in the clustered bed and from 0 to 3 in the continuous bed. Female mating success ranged from 0 to 5 in the clustered bed and from 0 to 2 in the continuous bed (Figure 2A). Male reproductive success ranged from 0 to 8 offspring in both shell bed types, while female reproductive success ranged from 0 to 8 in the clustered bed and ranged from 0 to 5 in the continuous bed (Figure 2B). For males, neither *I*, *I*s, *β*ss, nor *β′*_size_ differed significantly between the two shell bed types (Table 1; Figure 2C,D; Figure S2). For females, neither *I*s nor *β*ss differed significantly between the two shell bed types, but *I* and *β′*_size_ were marginally higher in the clustered bed relative to the continuous bed, though this did not reach statistical significance (Table 1; Figure 2C,D; Figure S2).

**TABLE 1:** Metrics of selection compared between shell bed types and sexes, presented following the recommendations outlined in Anthes et al. (2017). Note that to facilitate between‐study comparisons, Anthes et al. (2017) recommends that Bateman gradients, *β*ss, be calculated both with and without individuals that achieved zero mating success, and so we present both versions here. *NB* Fish with zero MS are likely young adults that are awaiting a breeding position in their social group. Including individuals with zero MS into the calculation of *β*ss (which is common practice when RS and MS are derived from genetic parentage data obtained from thoroughly sampled wild populations) is known to bias *β*ss upwards in females (i.e., the sex that receives fewer fitness returns with each additional mating, i.e., the shallower *β*ss) more so than males (Anthes et al. 2017).

Selection metrics	Clustered bed	Continuous bed	Permutation test results
Adult sex ratio (adult males: adult females)	*N* = 191 males, 239 females Ratio = 1:1.25	*N* = 90 males, 123 females Ratio = 1:1.37	NA
Reproductive success (RS): Number of offspring sired or produced per adult (average ± SD)	*RS*_(F)_ = 1.04 ± 1.53 (incl. 0 s) *RS*_(F)_ = 2.09 ± 1.57 (excl. 0 s) *RS*_(M)_ = 1.09 ± 1.75 (incl. 0 s) *RS*_(M)_ = 2.55 ± 1.86 (excl. 0 s)	*RS*_(F)_ = 1.02 ± 1.22 (incl. 0 s) *MS*_(F)_ = 1.85 ± 1.08 (excl. 0 s) *RS*_(M)_ = 1.31 ± 1.88 (incl. 0 s) *RS*_(M)_ = 2.81 ± 1.82 (excl. 0 s)	NA
Mating success (MS): Number of genetic mates per adult (average ± SD)	*MS*_(F)_ = 0.64 ± 0.77 (incl. 0 s) *MS*_(F)_ = 1.28 ± 0.61 (excl. 0 s) *MS*_(M)_ = 0.72 ± 1.0 (incl. 0 s) *MS*_(M)_ = 1.67 ± 0.86 (excl. 0 s)	*MS*_(F)_ = 0.65 ± 0.65 (incl. 0 s) *MS*_(F)_ = 1.18 ± 0.38 (excl. 0 s) *MS*_(M)_ = 0.68 ± 0.86 (incl. 0 s) *MS*_(M)_ = 1.45 ± 0.67 (excl. 0 s)	NA
Opportunity for selection, *I*	*I*_(F)_ = 2.14 *I*_(M)_ = 2.57	*I*_(F)_ = 1.43 *I*_(M)_ = 2.05	*P*_(F)_ = 0.067 *P*_(*M*)_ = 0.33
Opportunity for sexual selection, *I*s	*I*s_(F)_ = 1.47 *I*s_(M)_ = 1.95	*I*s_(F)_ = 1.01 *I*s_(M)_ = 1.61	*P*_(F)_ = 0.13 *P*_(*M*)_ = 0.43
Bateman gradient, *β*ss (slope ± SE)	*β*ss_(F)_ = 0.99 ± 0.044 (incl. 0 s) *β*ss_(M)_ = 1.02 ± 0.038 (incl. 0 s) *β*ss_(F)_ = 0.97 ± 0.114 (excl. 0 s) *β*ss_(M)_ = 1.08 ± 0.103 (excl. 0 s)	*β*ss_(F)_ = 0.99 ± 0.060 (incl. 0 s) *β*ss_(M)_ = 0.95 ± 0.065 (incl. 0 s) *β*ss_(F)_ = 0.95 ± 0.187 (excl. 0 s) *β*ss_(M)_ = 0.83 ± 0.180 (excl. 0 s)	*P*_(F)_ = 0.98 *P*_(*M*)_ = 0.53 *P*_(F)_ = 0.91 *P*_(*M*)_ = 0.27
Selection gradient for body size, *β′*_size_	*β′*_size (F)_ = 0.30 *β′*_size (M)_ = 0.52	*β′*_size (F)_ = 0.021 *β′*_size (M)_ = 0.54	*P*_(F)_ = 0.065 *P*_(*M*)_ = 0.90

### 3.2 No Clear Differences in Dispersal Patterns Between Shell Beds

**FIGURE 3:**
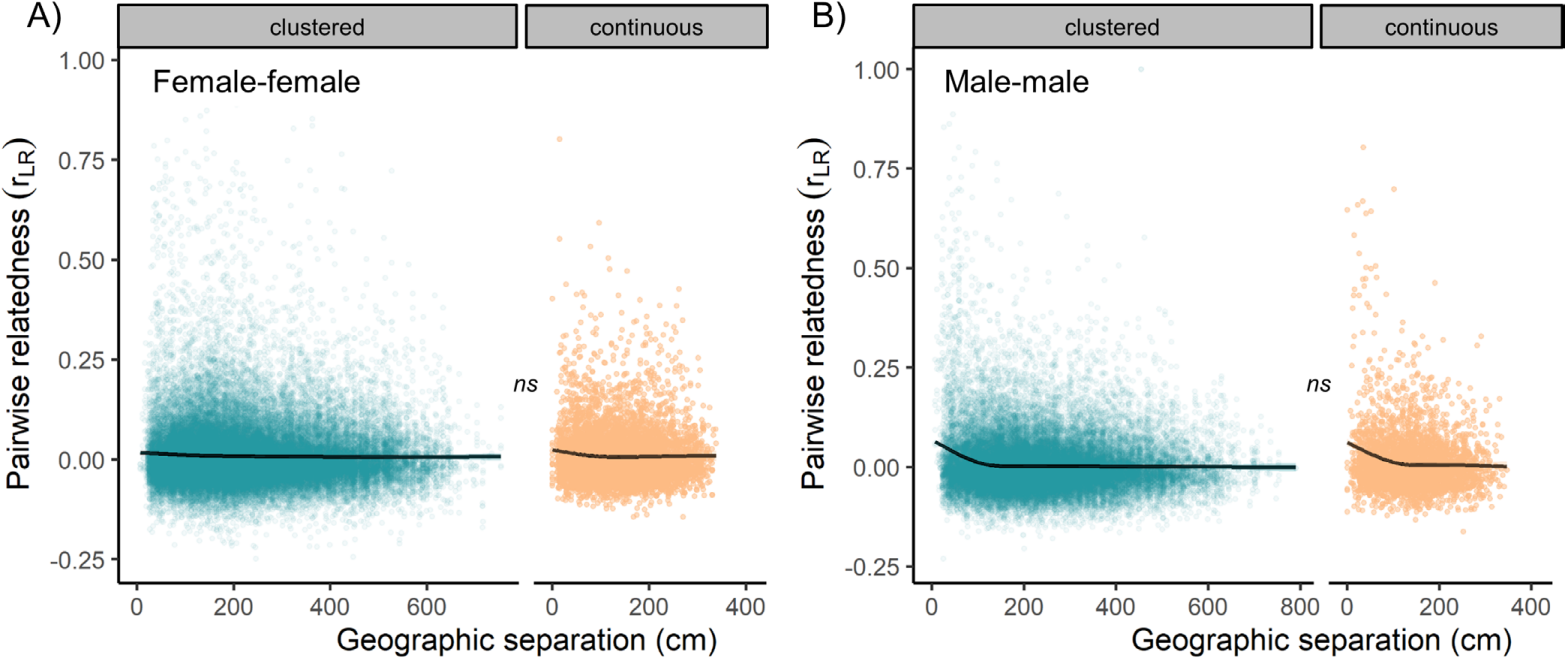
Genetic relatedness of same‐sex adults over geographic separation on either the clustered or continuous shell bed. (A) Female–female relatedness. (B) Male–male relatedness. Plots show GAM fits and untransformed data points.

Female–female relatedness coefficients declined very shallowly with geographic separation in the continuous shell bed (GAMM, edf = 2.60, *F* = 3.71, *p* = 0.011), and this pattern was not significantly different from that in the clustered shell bed (edf = 1.02, *F* = 0.17, *p* = 0.70, Figure 3A). Male–male relatedness coefficients declined with geographic separation in the continuous shell bed (GAMM, edf = 3.51, *F* = 7.09, *p* < 0.0001), and this pattern was also not significantly different from that in the clustered shell bed (edf = 2.02, *F* = 0.34, *p* = 0.77, Figure 3B).

### 3.3 Threatening Heterospecifics Are Present for More Time at Continuous Shell Beds Relative to Clustered Shell Beds

**FIGURE 4:**
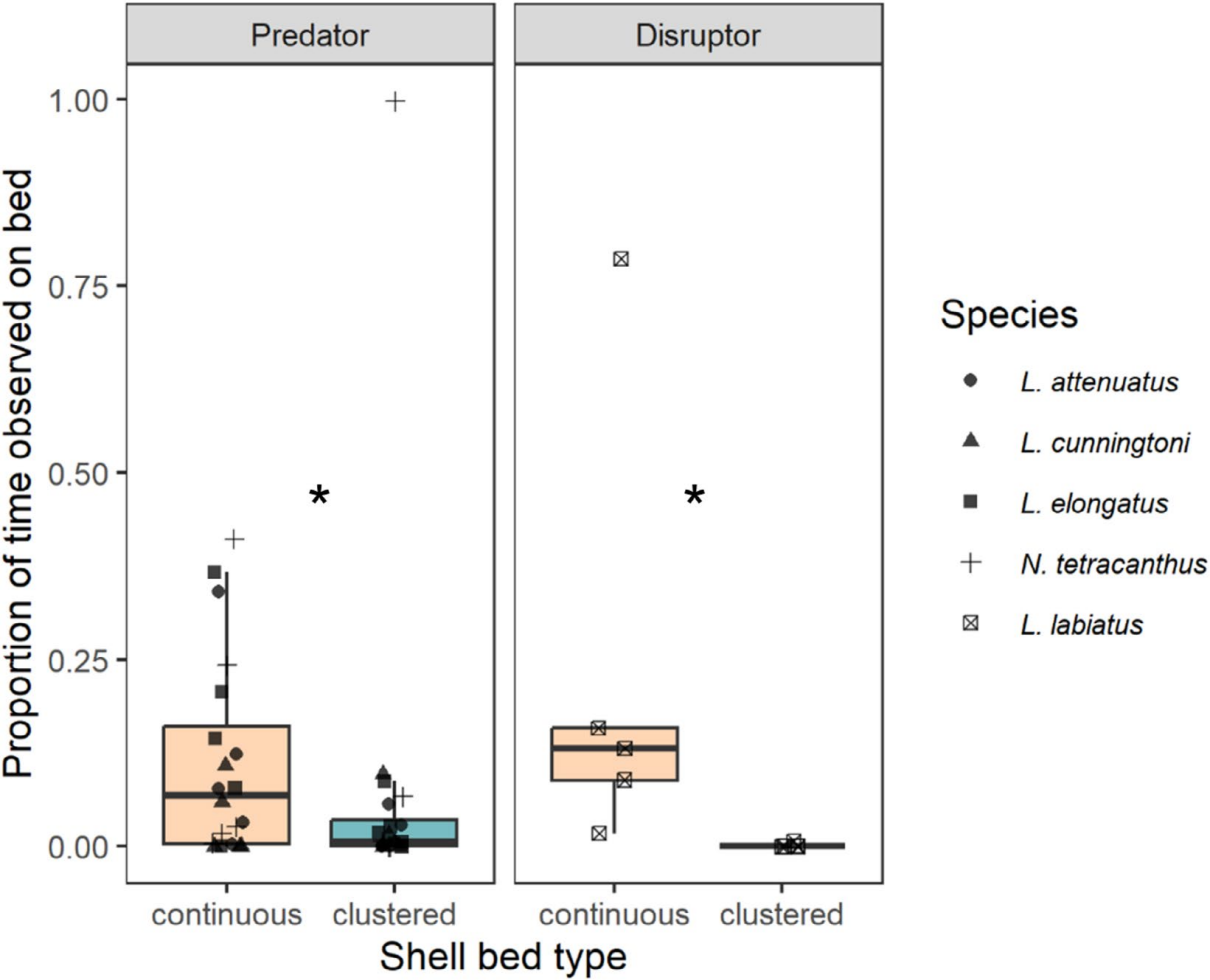
Proportion of time that heterospecifics were observed over either continuous or clustered shell beds. *L. attenuatus* , *L. elongatus* , *L. cunningtoni* , and *N. tetracanthus* are predators of *N. multifasciatus* , while *L. labiatus* is a species whose foraging behavior disrupts the arrangement of shells on the shell bed, which can adversely affect shell‐dwellers like *N. multifasciatus* . * denote significant contrasts at *p* < 0.05.

Overall, predators were present for more time at continuous shell beds than clustered beds (11.2% vs. 7.1% of the observed time, LMM emmeans, est. ± SE = 1.64 ± 0.72, *t*‐ratio = 2.26, *p* = 0.029, Figure 4). *L. labiatus* were also present for significantly more time at continuous beds than clustered beds (23.6% vs. 0.2% of the observed time; est. ± SE = 5.15 ± 1.45, *t*‐ratio = 3.56, *p* = 0.0009, Figure 4).

### 3.4 Adults Grow to Become Larger in Clustered Shell Beds Relative to Continuous Shell Beds

**FIGURE 5:**
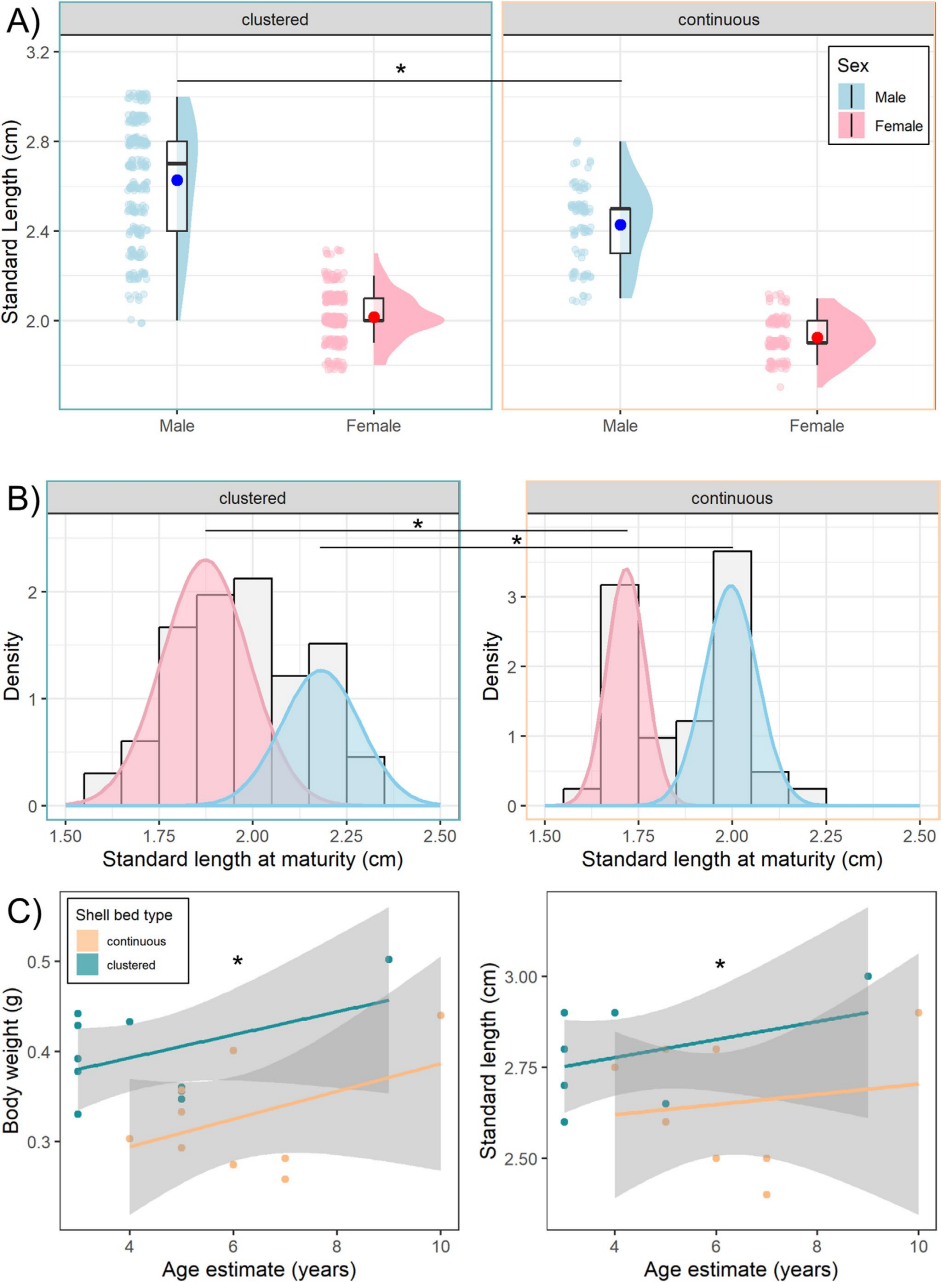
(A) Standard lengths of adult males and females from each shell bed type (*N*_total_ = 772 adults). Data are presented as raw datapoints (slightly jittered to improve visibility) to the left of boxplots as well as density histograms. Box plots show group means (darker dots), medians (horizontal bars), the interquartile range (boxes) and the extent of the data no more than 1.5 times the interquartile range above the box (whiskers). (B) Results of Gaussian mixture models used to estimate size‐at‐maturity for males and females in the clustered and continuous shell beds separately (*N*_total_ = 107 juveniles). Grey histograms show the body sizes for the sampled *N. multifasciatus* with faint banding patterns on their bodies (denoting juveniles transitioning to maturity). Sex‐specific estimated probability density functions are shown for each shell bed region (pink for females, blue for males). (C) Size versus age relationships for male *N. multifasciatus* from clustered and continuous shell beds (*N*_total_ = 19 males). Left panel shows body weight, while right panel shows standard length. * denote significant contrasts at *p* < 0.05.

Adult males from clustered shell beds were on average larger than males from continuous shell beds (emmeans, LMM, est. ± SE = 0.16 ± 0.047, *z* = 3.49, *p* = 0.0029), but females were not (est. ± SE = 0.053 ± 0.045, *z* = 1.17, *p* = 0.65, Figure 5A). The calculated 95% confidence interval for the 90th percentile of SL for males was 2.9–3.0 cm in the clustered shell bed and 2.6–2.7 cm in the continuous shell bed. For females, this was 2.1–2.2 cm in the clustered shell bed and 2.0–2.1 cm in the continuous shell bed. The lack of overlap in the 95% CIs between shell beds suggests that the upper body size limits for males and females are larger in the clustered shell beds relative to the continuous shell beds.

### 3.5 Fish Mature at Larger Body Sizes on Clustered Shell Beds Relative to Continuous Shell Beds

On the clustered shell beds, male size‐at‐maturity was estimated to be 2.18 ± 0.11 cm (mean ± std. dev, 95% CI range: 2.14–2.23 cm), and female size‐at‐maturity 1.87 ± 0.12 cm (95% CI range: 1.84–1.91 cm, Figure 5B). On the continuous shell bed, male size‐at‐maturity was estimated to be 2.00 ± 0.07 cm (95% CI range: 1.97–2.02 cm), and female size‐at‐maturity 1.72 ± 0.05 cm (95% CI range: 1.69–1.74 cm, Figure 5B). 95% confidence intervals for size‐at‐maturity did not overlap between the two shell bed types for both males and females.

### 3.6 Adult Males Are Larger for Their Ages in the Clustered Shell Bed Than the Continuous Shell Bed

The estimated ages of the males in our sample ranged from 3 to 10 years old (Figure 5C). Across the ages captured within our sample, male body weight increased with age (LM, est. ± SE = 0.014 ± 0.006, *z* = 2.16, *p* = 0.031) but standard length did not increase significantly (est. ± SE = 0.020 ± 0.019, *z* = 1.06, *p* = 0.29). Males from the clustered shell beds were larger for their age than males from the continuous shell bed, both in terms of body weight (est. ± SE = 0.095 ± 0.025, *z* = 3.77, *p* = 0.0002) and standard length (est. ± SE = 0.17 ± 0.074, *z* = 2.32, *p* = 0.021, Figure 5C).

## 4 Discussion

Spatial and temporal heterogeneity in environmental conditions is known to affect the strength and direction of selection on traits (Cornwallis and Uller 2010; Miller and Svensson 2014), and a powerful method for uncovering the environmental moderators of evolution is to compare the strength of selection among natural populations that differ in a putative ecological driver of selection (Siepielski et al. 2013). Typically, organisms have limited control over how resources are distributed throughout their environment, and this has shaped the classical view that the causes of selection stem primarily from the external environment with little opportunity for organisms to actively modify the selective regimes they experience (Day et al. 2003). The shell beds of Lake Tanganyika therefore offer a unique opportunity to investigate the eco‐evolutionary effects that niche construction (i.e., the generation of shell clusters) may have on the shell‐dwelling fishes that live in these areas. This is because shell beds like the one at our study site are naturally subdivided into replicate sub‐regions, in which resources are either clumped or homogeneous due to the niche constructing activities of *N. multifasciatus* (Figure 1). The natural differences in resource heterogeneity between clustered and continuous shell beds additionally allow us to explore multiple additional effects that resource distributions have on wild populations.

Because *N. multifasciatus* changes the lake floor from a (nearly) homogenous mixture of sand and shells into a clumped distribution of shells, we first investigated whether this niche construction would increase the fish's own environmental potential for polygyny (*sensu* Emlen and Oring 1977) and thereby elevate the strength of sexual selection in clustered shell beds relative to continuous shell beds. In contrast to our predictions, we did not detect evidence of stronger selection in clustered shell beds relative to continuous shell beds. While the opportunity for selection, *I*, and the opportunity for sexual selection, *I*s, were higher in the clustered bed relative to the continuous bed, none of our calculated selection metrics differed significantly between the two focal shell bed patches. In *N. multifasciatus* , dominant males control territories that contain critical shell resources that females need to reproduce (Bose, Dabernig‐Heinz, et al. 2022; Bose, Koch, et al. 2022; Jordan et al. 2016). Yet, despite the fish forming shells into clumps in the clustered shell bed and surrounding them with sand walls, dominant males appeared to be no better at monopolizing access to their shells and receiving their reproductive benefits when compared to large males on the continuous shell bed. We found weak evidence, however, that body size in females may be under stronger positive selection in the clustered shell bed compared to the continuous shell bed (although the difference was not significant). Like males, female *N. multifasciatus* are also aggressive and they defend sub‐territories within the larger territories of dominant males (Bose et al. 2021, 2023; Gübel et al. 2021), potentially benefiting from the clumped distribution of shells in this habitat. Nevertheless, the overall lack of a clear relationship between resource clumping and variance‐based metrics of selection is consistent with some previous studies that have similarly attempted to look at within‐population variation in selection in relation to resource distributions. For example, in a field experiment by Wong et al. (2018) on sand goby, *Pomatoschistus minutus* , artificial nest sites placed either in aggregated or sparse distributions did not ultimately result in clear differences in the opportunity for selection *I* for nesting males, though selection gradients on body size did differ initially during the early breeding season when females were beginning to arrive at nests. Similarly, Reichard et al. (2009) conducted a mesocosm experiment with bitterling, *Rhodeus amarus* , in which oviposition sites were either clumped or sparsely distributed. As with our current study, their opportunity for selection *I* was generally higher in the clumped condition but did not differ significantly between treatments; however, the authors of that study did detect differences in selection gradients on certain phenotypic traits like gonad size.

A notable difference between our study and these previous studies is that our clumped resources were also less dense when averaged across the landscape (the shells in the continuous bed were both denser and more homogenously distributed). How this translates to differences in population density is an important question. There is ample evidence from birds that population density can increase levels of reproductive competition and sexual selection (Kokko and Rankin 2006; Schlicht et al. 2015; Westneat and Sherman 1997), often with competition being most intense among near neighbours (e.g., Canal et al. 2012). While the density of exposed shells is higher in continuous shell beds relative to clustered shell beds, it is still unclear whether densities of resident *N. multifasciatus* , or the spacing between their social groups, differ meaningfully. A *post hoc* calculation incorporating the number of fish sampled and the areas of our two focal shell bed patches revealed that population density was 55.3 fish/m^2^ in the clustered shell bed and 63.0 fish/m^2^ in the continuous shell bed (28.5 fish/m^2^ and 29.2 fish/m^2^ respectively, when considering adults only). Thus, similarity in population density between the shell beds may in part explain similarities in selection that we calculated. It will also be valuable for future studies to evaluate group size, composition, and spatial positioning in continuous shell beds, which we did not do here. This will be useful to clarify whether individuals or social groups maintain the same spacing between one another regardless of the presence of sand walls.

Male and female dispersal patterns did not differ clearly between either habitat. Previously, Bose, Koch, et al. (2022) revealed that male *N. multifasciatus* living in a clustered shell bed displayed elevated relatedness to other males within a 1–2 m radius, and this difference was greater compared to female–female relatedness on the same spatial scale. Such a pattern is indicative of female‐biased dispersal, which in *N. multifasciatus* is likely driven by a combination of factors resulting in females paying fewer costs than males while dispersing (Bose, Koch, et al. 2022). Here, we show a similar pattern of sex‐specific dispersal in the continuous bed as in the clustered bed, including low female–female relatedness throughout both shell beds, including at short distances, and a steeper decrease in male–male relatedness over a spatial scale of 1–2 m. Therefore, a greater density of shelter resources in the continuous shell bed did not clearly facilitate dispersal over longer distances on average than in the clustered shell bed. This is consistent with both shell beds being saturated habitats in which many shells are already occupied by territorial residents (Bose et al. 2024) that might similarly resist dispersers taking up temporary shelter. Alternatively, enhanced dispersal opportunities in the continuous bed afforded by more ‘stepping stone’ refuges might be counteracted by other ecological constraints such as predation pressure.

Continuous shell beds attracted more activity from predatory or disruptive heterospecifics than clustered shell beds, and with comparable population densities in each shell bed type, we suggest that predation pressures are likely to be higher on continuous shell beds. Predators may be attracted to continuous shell beds because without the sand digging activities of *N. multifasciatus* , fewer shells become buried. Shells provide shelter for a range of organisms (Salzburger et al. 2014), and so continuous shell beds may present more rewarding foraging patches for predatory species, which can directly or indirectly threaten the *N. multifasciatus* that live there. Particularly notable was the increased presence of *L. labiatus* on continuous beds relative to clustered beds, where they were often observed overturning shells via their suction feeding, a disturbance that was then also amplified by frequent coalitions of *L. labiatus* with schools of *Lamprologus callipterus* , forming foraging groups that sift through the substrata together (as described in Yuma 1994). Our parentage data also lend support to the idea that mortality rates are naturally higher on continuous shell beds. While we could detect at least one parent in the clustered shell bed for 92% of offspring living there (70.8% had a detected mother, 59.2% had a detected father), we could only detect this for 73% of the offspring living in the continuous shell bed (49.4% had a detected mother, 46.6% had a detected father). Given that both shell bed patches were thoroughly sampled, and that they were each bordered by a stretch of open sand constituting a dispersal barrier, this suggests that undetected parents had died prior to our sampling.

Consistent with predictions from life‐history theory (Bonduriansky et al. 2008; Magnhagen 1991), we detected life history differences between the *N. multifasciatus* living in each shell bed type. Fish from the clustered shell beds mature at larger body sizes, are larger at a given age (starting at 3 years of age for males in our dataset), and ultimately grow to be larger overall than those from the continuous shell bed. The life history strategy in the clustered shell bed appears to favour investment into somatic growth at the expense of later maturation, whereas the strategy in the continuous shell bed favours reduced growth in return for earlier maturation. Previous work has similarly shown life history differences within or between closely situated populations in relation to resource availability. For example, Japanese fluvial sculpin, *Cottus pollux* , living on either side of a weir in a river displayed earlier maturation and shorter lifespans in the area with high nest abundance (Natsumeda et al. 2013). Takahashi (2008) also investigated two populations of freshwater goby, *Tridentiger brevispinis* , from the same lake that differed in nest site availability and showed that when nest sites were scarce males matured later and grew larger than when nest sites were abundant. Ultimately, when individuals can competitively exclude one another from critical resources, life history expression can shift towards later maturation and more somatic growth. We suggest that our life history patterns of later maturation and heavier investment into somatic growth in clustered shell beds are consistent with a more competitive reproductive environment. Our finding that fish on the continuous shell bed matured at smaller body sizes (and presumably at younger ages) is also in line with the well‐established prediction that investment into reproduction at the expense of growth ought to be favoured under higher predation pressure (when there are no alternatives such as growing to a body size that escapes predation risk, Magnhagen 1991). Food availability is unlikely to differ greatly between the two habitats as *N. multifasciatus* forage primarily by snatching plankton from the water column, rather than grazing or hunting on the lake floor (Kohler 1998; Lein and Jordan 2021). It remains to be tested whether the differences in life history that we uncovered are due to phenotypic plasticity or adaptive genetic change, as the definition of niche construction—as a force for evolutionary change—requires that recipient populations undergo genetic change because of environmental modifications. Our mapping of particular shell bed regions between successive years suggests that the overall structure of the shell bed is rather invariant across behavioural and generational timescales (Figure S1). And while patches of continuous and clustered shell beds can be separated by mere meters in the wild, these distances also constitute significant dispersal barriers for *N. multifasciatus* (Bose, Dabernig‐Heinz, et al. 2022; Bose, Koch, et al. 2022). Overall, this sets the stage for successive generations to inherit their parents' modified or unmodified environments, allowing adaptation, plasticity, or a combination of both to underlie our life history differences, a question that common garden experiments will be needed to answer (Matthews et al. 2014).

In this comprehensive study, we combined field and genetic data to evaluate whether conspicuous differences in resource heterogeneity in modifiable and unmodifiable regions of shell bed are associated with measurable differences in metrics of selection as predicted by the environmental potential for polygyny (Emlen and Oring 1977), as well as in patterns of dispersal, predation, and life history. Despite conspicuous differences in resource density and distribution between the clustered and continuous shell beds, we detected no clear differences in metrics of selection or dispersal. However, we did detect striking predation and life history differences between the shell bed types. It should be noted that although we compared selection and dispersal between the continuous and clustered shell bed 4 years apart, a timeframe over which lake conditions may have changed subtly, we detected no clear differences in these endpoints. However, when we did detect differences between the shell bed types in predation and life history, these were from comparisons where year effects were accounted for. Overall, we highlight the utility of finding natural experiments where populations are subdivided into replicates of differing resource arrangements to explore the multifaceted nature of resource heterogeneity and niche construction.

## Author Contributions

A.P.H.B. conceived the research. A.P.H.B., B.D., and L.K. conducted the field work. A.P.H.B. conducted the genotyping analyses with assistance from J.G. and K.M.S. Funding was secured by A.P.H.B., A.J., and K.M.S. Statistical and parentage analyses were conducted by A.P.H.B., J.H., and K.M.S. A.P.H.B. wrote the manuscript with input from all co‐authors. All authors read and approved the final manuscript.

## Conflicts of Interest

The authors declare no conflicts of interest.

## Supporting information


**Data S1:** mec70145‐sup‐0001‐Supinfo.pdf.

## Data Availability

The data that support the findings of this study are openly available in OSF at https://doi.org/10.17605/OSF.IO/PES3R.
