# Supplementary material for: Habitat Differences in Resource Density and Distribution Affect Ecology and Life History of a Landscape‐Modifying Fish
Source: Mol Ecol. 2025 Oct 13;34(22):e70145. doi: 10.1111/mec.70145 (PMC12617062; doi:10.1111/mec.70145)
Supplement: Supplementary file 1 — Data S1: mec70145‐sup‐0001‐Supinfo.pdf. [file MEC-34-e70145-s001.pdf]

MOLECULAR ECOLOGY

Supplemental Information for:

Habitat differences in resource density and distribution affect ecology and life history of a landscape-modifying fish

Aneesh P. H. Bose, Boyd Dunster, Jonathan Henshaw, Lukas Koch, Jacqueline Grimm,
Kristina M. Sefc, Alex Jordan

Table of Contents:

**Supplementary Materials Figure S1**	Page 2
**Supplementary Materials Figure S2**	Page 3
**Supplementary Materials Table S1**	Page 4
**Supplementary information for parentage analysis conducted on clustered shell bed (2019).**	Page 5
**Supplementary Materials Table S2**	Page 6
**Supplementary information for parentage analysis conducted on continuous shell bed (2023).**	Page 7
**Supplementary references**	Page 8

MOLECULAR ECOLOGY

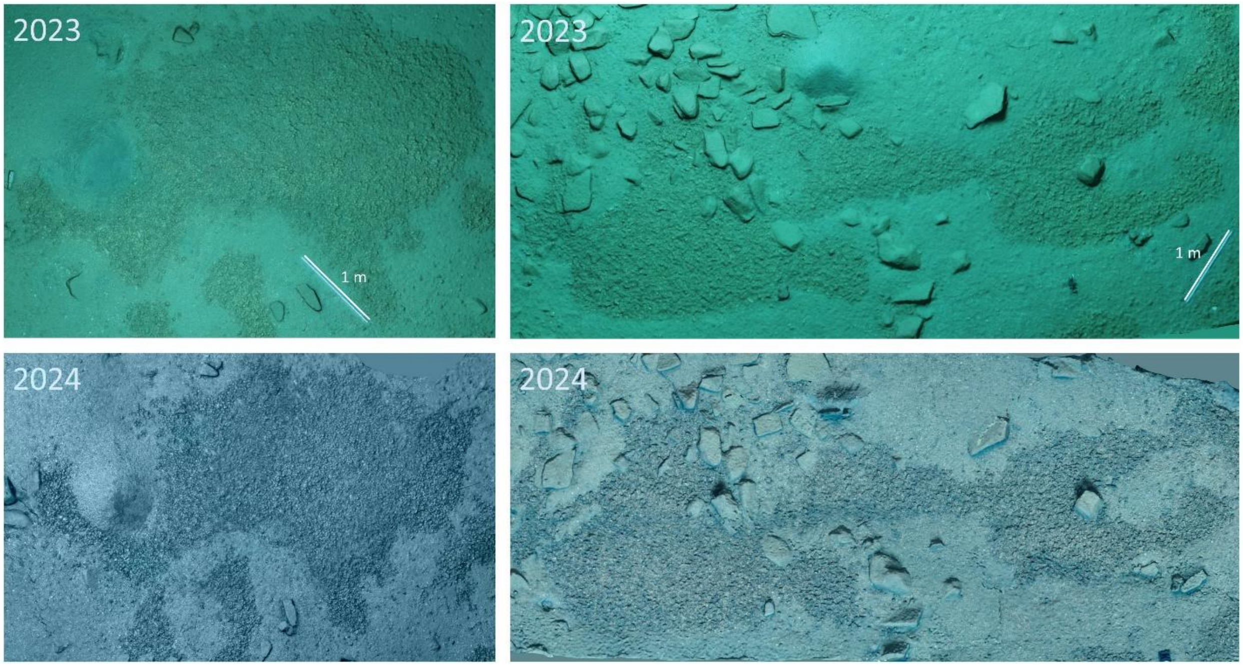

Supplementary Materials Figure S1: Two continuous shell beds mapped one year apart using Structure-from-Motion (SfM) photogrammetry. Left panels show the continuous shell bed sampled for population genetics in 2023, while the right panels show a continuous shell bed that was untouched. SfM mapping of these locations was derived from video footage (Go Pro Hero 10 Black, 2.7k, linear FOV) from swim-overs performed in spring 2023 and again in spring 2024.

MOLECULAR ECOLOGY

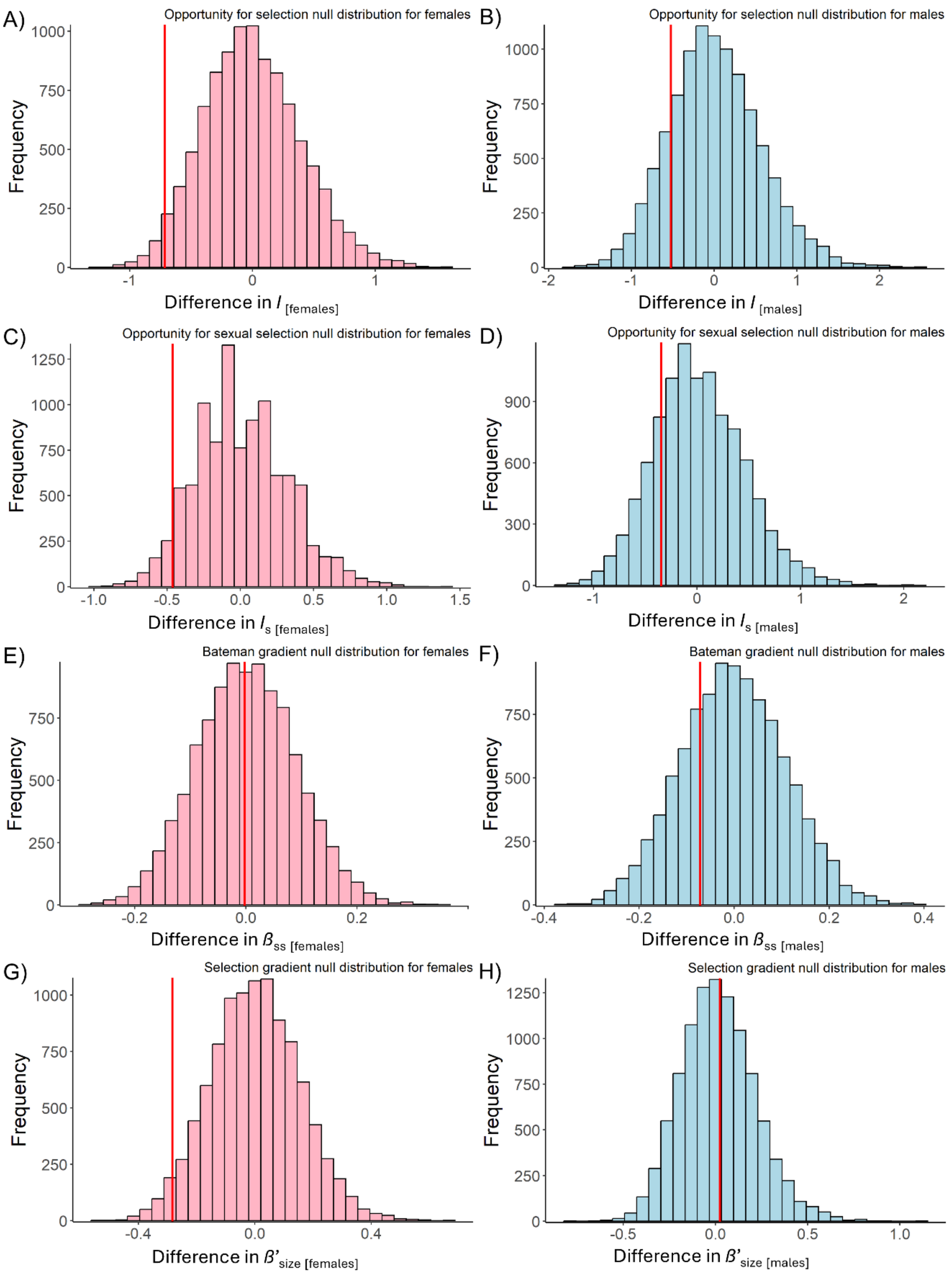

Supplementary Materials Figure S2: Results of permutation tests comparing metrics of selection and sexual selection between the clustered and continuous shell bed for males (blue)

MOLECULAR ECOLOGY

and females (pink). Red vertical line is our observed differences in selection metrics between the two shell bed types.

Supplementary Materials Table S1: Marker polymorphism in reference population (238 fish) of 20 microsatellites used to assign parentage and calculate relatedness in the clustered shell bed (data collected in 2019). k: Number of alleles. N: Number of individuals genotyped at the particular locus. H_{Obs}: Observed Heterozygosity (proportion of heterozygotes at this locus). H_{Exp}: Expected heterozygosity (expected proportion of heterozygotes given allele frequencies). HW: Adherence to Hardy-Weinberg Equilibrium, tested in CERVUS using a Bonferroni correction (Bonferroni corrected α = 0.0025). Shading denotes separation of different multiplexes.

Locus	k	N	H_{Obs}	H_{Exp}	HW *P*-value	Conc. in primer mix (pmol/µL)	Reference
Multiplex 1							
Pmv17	19	233	0.906	0.912	0.50	0.5	(Crispo et al., 2007)
UNH890	6	232	0.414	0.436	0.73	1.0	(Carleton et al., 2002)
UNH908	25	235	0.843	0.875	0.36	3.0	(Carleton et al., 2002)
Gm634	15	234	0.799	0.818	0.42	1.0	(B. Y. Lee et al., 2005)
Ppun9	21	233	0.674	0.748	0.02	0.5	(Taylor et al., 2002)
Hchi59	17	232	0.845	0.864	0.52	1.0	(Maeda et al., 2008)
UNH216	11	232	0.603	0.584	0.83	4.0	(W. J. Lee & Kocher, 1996)
UME002	7	228	0.61	0.627	0.44	4.0	(Parker & Kornfield, 1996)
Multiplex 2							
Pmv3	31	237	0.768	0.775	0.04	1.0	(Crispo et al., 2007)
GM264	17	234	0.85	0.859	0.42	4.0	(B. Y. Lee et al., 2005)
Ppun5	23	233	0.695	0.722	0.19	3.0	(Taylor et al., 2002)
TmoM13	25	234	0.829	0.907	0.44	4.0	(Zardoya et al., 1996)
TmoM25	4	231	0.732	0.671	0.55	2.0	(Zardoya et al., 1996)
Hchi36	4	230	0.539	0.559	0.44	1.0	(Maeda et al., 2008)
UME003	17	232	0.897	0.869	0.56	2.0	(Parker & Kornfield, 1996)
Multiplex 3							
TmoM11	7	234	0.667	0.677	0.87	1.5	(Zardoya et al., 1996)
UNH2075	19	233	0.773	0.77	0.23	2.5	(Albertson et al., 2003)
NP101	18	232	0.81	0.749	0.02	3.5	(Brandtmann et al., 1999)
Pzeb4	8	232	0.612	0.61	0.98	2.0	(Van Oppen et al., 1997)
UNH974	33	219	0.863	0.926	0.60	4.0	(Carleton et al., 2002)

Supplementary information for parentage analysis conducted on clustered shell bed (2019).

Parentage of offspring was assigned based on maximum likelihood simulations in the software CERVUS using the following simulation parameters:

Input parameters	
Number of offspring:	1000
Number of candidate mothers:	306*
Proportion of candidate mothers sampled:	0.80
Number of candidate fathers:	245*
Proportion of candidate fathers sampled:	0.80
Number of parent pairs:	74970
Proportion of loci typed:	0.95
Proportion of loci mistyped:	0.05
Error rate in likelihood calculations:	0.05
Minimum number of typed loci:	15
Output parameters	
Confidence determined using:	Delta
Relaxed confidence level:	80.0%
Strict confidence level:	95.0%

* Here, the number of candidate mothers and fathers in the simulations represented our estimates of the total number of adult females and males in the population if we had sampled only 80% of them. While sampling the clustered shell bed, we captured 239 adult females (plus 6 juvenile females with developing banding patterns on the cusp of maturation and so were included here as well) and 191 adult males (plus 5 juvenile males on the cusp of maturation). Hence, the number of candidate mothers is 306 (245/0.8) and the number of candidate fathers is 245 (196/0.8).

MOLECULAR ECOLOGY

Supplementary Materials Table S2: Marker polymorphism in reference population (of 90 fish) of 20 microsatellites used to assign parentage and calculate relatedness in the continuous shell bed (data collected in 2023). k: Number of alleles. N: Number of individuals genotyped at the particular locus. H_{Obs}: Observed Heterozygosity (proportion of heterozygotes at this locus). H_{Exp}: Expected heterozygosity (expected proportion of heterozygotes given allele frequencies). HW: Adherence to Hardy-Weinberg Equilibrium, tested in CERVUS using a Bonferroni correction (Bonferroni corrected α = 0.0025). Shading denotes separation of different multiplexes.

Locus	K	N	H_{Obs}	H_{Exp}	HW *P*-value	Conc. in primer mix (pmol/µL)	Reference
Multiplex 1							
Pmv17	19	90	0.867	0.919	0.75	0.5	(Crispo et al., 2007)
UNH890	5	90	0.522	0.516	0.34	1.0	(Carleton et al., 2002)
UNH908	20	90	0.944	0.905	0.14	3.0	(Carleton et al., 2002)
Gm634	9	90	0.744	0.751	0.79	1.0	(B. Y. Lee et al., 2005)
Ppun9	15	90	0.767	0.777	0.24	0.5	(Taylor et al., 2002)
Hchi59	14	90	0.922	0.88	0.32	1.0	(Maeda et al., 2008)
UNH216	10	90	0.556	0.558	0.74	4.0	(W. J. Lee & Kocher, 1996)
UME002	6	90	0.589	0.654	0.41	4.0	(Parker & Kornfield, 1996)
Multiplex 2							
Pmv3	24	90	0.767	0.763	0.71	1.0	(Crispo et al., 2007)
GM264	12	90	0.889	0.847	0.75	4.0	(B. Y. Lee et al., 2005)
Ppun5	13	90	0.722	0.744	0.48	3.0	(Taylor et al., 2002)
TmoM13	18	90	0.856	0.87	0.25	4.0	(Zardoya et al., 1996)
TmoM25	4	90	0.678	0.658	0.38	2.0	(Zardoya et al., 1996)
Hchi36	4	90	0.533	0.537	0.68	1.0	(Maeda et al., 2008)
UME003	15	90	0.778	0.816	0.038	2.0	(Parker & Kornfield, 1996)
Multiplex 3							
TmoM11	7	90	0.633	0.657	0.93	1.5	(Zardoya et al., 1996)
UNH2075	15	90	0.689	0.758	0.26	2.5	(Albertson et al., 2003)
NP101	16	90	0.789	0.791	0.11	3.5	(Brandtmann et al., 1999)
Pzeb4	7	90	0.644	0.577	0.42	2.0	(Van Oppen et al., 1997)
UNH974	28	90	0.833	0.892	0.0035	4.0	(Carleton et al., 2002)

MOLECULAR ECOLOGY

Supplementary information for parentage analysis conducted on continuous shell bed (2023).

Parentage of offspring was assigned based on maximum likelihood simulations in the software CERVUS using the following simulation parameters:

Input parameters	
Number of offspring:	1000
Number of candidate mothers:	154*
Proportion of candidate mothers sampled:	0.80
Number of candidate fathers:	113*
Proportion of candidate fathers sampled:	0.80
Number of parent pairs:	17784
Proportion of loci typed:	0.95
Proportion of loci mistyped:	0.05
Error rate in likelihood calculations:	0.05
Minimum number of typed loci:	15
Output parameters	
Confidence determined using:	Delta
Relaxed confidence level:	80.0%
Strict confidence level:	95.0%

* Here, the number of candidate mothers and fathers in the simulations represented our estimates of the total number of adult females and males in the population if we had sampled only 80% of them. While sampling the continuous shell bed, we captured 123 adult females and 90 adult males. Hence, the number of candidate mothers is 154 (123/0.8) and the number of candidate fathers is 113 (90/0.8).

Supplementary references

Albertson, R. C., Streelman, J. T., & Kocher, T. D. (2003). Directional selection has shaped the oral jaws of Lake Malawi cichlid fishes. *Proceedings of the National Academy of Sciences of the United States of America*, *100*(9), 5252–5257. https://doi.org/10.1073/pnas.0930235100

Brandtmann, G., Scandura, M., & Trillmich, F. (1999). Female-female conflict in the harem of a snail cichlid (*Lamprologus ocellatus*): Behavioural interactions and fitness consequences. *Behaviour*, *136*(9), 1123–1144.

Carleton, K. L., Streelman, J. T., Lee, B. Y., Garnhart, N., Kidd, M., & Kocher, T. D. (2002). Rapid isolation of CA microsatellites from the tilapia genome. *Animal Genetics*, *33*(2), 140–144. https://doi.org/10.1046/j.1365-2052.2002.00817.x

Crispo, E., Hagen, C., Glenn, T., Geneau, G., & Chapman, L. J. (2007). Isolation and characterization of tetranucleotide microsatellite markers in a mouth-brooding haplochromine cichlid fish (*Pseudocrenilabrus multicolor victoriae*) from Uganda. *Molecular Ecology Notes*, *7*(6), 1293–1295. https://doi.org/10.1111/j.1471-8286.2007.01859.x

Lee, B. Y., Lee, W. J., Streelman, J. T., Carleton, K. L., Howe, A. E., Hulata, G., Slettan, A., Stern, J. E., Terai, Y., & Kocher, T. D. (2005). A second-generation genetic linkage map of tilapia (Oreochromis spp.). *Genetics*, *170*(1), 237–244. https://doi.org/10.1534/genetics.104.035022

Lee, W. J., & Kocher, T. D. (1996). Microsatellite DNA markers for genetic mapping in *Oreochromis niloticus*. *Journal of Fish Biology*, *49*(1), 169–171. https://doi.org/10.1006/jfbi.1996.0145

Maeda, K., Takeshima, H., Mizoiri, S., Okada, N., Nishida, M., & Tachida, H. (2008). Isolation and characterization of microsatellite loci in the cichlid fish in Lake Victoria, *Haplochromis chilotes*. *Molecular Ecology Resources*, *8*(2), 428–430. https://doi.org/10.1111/j.1471-8286.2007.01981.x

Parker, A., & Kornfield, I. (1996). Polygynandry in *Pseudotropheus zebra*, a cichlid fish from Lake Malawi. *Environmental Biology of Fishes*, *47*(4), 345–352. https://doi.org/10.1007/BF00005049

Taylor, M. I., Meardon, F., Turner, G., Seehausen, O., Mrosso, H. D. J., & Rico, C. (2002). Characterization of tetranucleotide microsatellite loci in a Lake Victorian, haplochromine cichlid fish: A *Pundamilia pundamilia* x *Pundamilia nyererei* hybrid. *Molecular Ecology Notes*, *2*(4), 443–445. https://doi.org/10.1046/j.1471-8286.2002.00272.x

Van Oppen, M. J. H., Rico, C., Deutsch, J. C., Turner, G. F., & Hewitt, G. M. (1997). Isolation and characterization of microsatellite loci in the cichlid fish *Pseudotropheus zebra*. *Molecular Ecology*, *6*(4), 387–388. https://doi.org/10.1046/j.1365-294X.1997.00188.x

Zardoya, R., Vollmer, D. M., Craddock, C., Streelman, J. T., Karl, S., & Meyer, A. (1996). Evolutionary conservation of microsatellite flanking regions and their use in resolving the phylogeny of cichlid fishes (Pisces: Perciformes). *Proceedings of the Royal Society B: Biological Sciences*, *263*(1376), 1589–1598. https://doi.org/10.1098/rspb.1996.0233
